# Effect of ensiled mulberry leaves and sun-dried mulberry fruit pomace on the fecal bacterial community composition in finishing steers

**DOI:** 10.1186/s12866-017-1011-9

**Published:** 2017-04-21

**Authors:** Yan Li, Qingxiang Meng, Bo Zhou, Zhenming Zhou

**Affiliations:** 1 State Key Laboratory of Animal Nutrition, Beijing, 100193 People’s Republic of China; 2 College of Animal Science and Technology, China Agricultural University, Beijing, 100193 People’s Republic of China

**Keywords:** Fecal bacteria community composition, Sequencing, Ensiled mulberry leaves (EML), Sun-dried mulberry fruit pomace (SMFP)

## Abstract

**Background:**

Here, we aimed to investigate the effects of ensiled mulberry leaves (EML) and sun-dried mulberry fruit pomace (SMFP) on fecal bacterial communities in Simmental crossbred finishing steers. To this end, the steers were reared on a standard TMR diet, standard diet containing EML, and standard diet containing SMFP. The protein and energy levels of all the diets were similar. Illumina MiSeq sequencing of the V4 region of the *16S* rRNA gene and quantitative real-time PCR were used to analyze and detect the fecal bacterial community.

**Results:**

Most of the sequences were assigned to *Firmicutes* (56.67%) and *Bacteroidetes* (35.90%), followed by *Proteobacteria* (1.87%), *Verrucomicrobia* (1.80%) and *Tenericutes* (1.37%). The predominant genera were *5-7 N15* (5.91%), *CF231* (2.49%), *Oscillospira* (2.33%), *Paludibacter* (1.23%) and *Akkermansia* (1.11%). No significant differences were observed in the numbers of *Firmicutes* (*p* = 0.28), *Bacteroidetes* (*p* = 0.63), *Proteobacteria* (*p* = 0.46), *Verrucomicrobia* (*p* = 0.17), and *Tenericutes* (*p* = 0.75) populations between the treatment groups. At the genus level, genera classified with high abundance (more than 0.1%) belonged primarily to *Bacteroidetes* and *Firmicutes*. Furthermore, no differences were observed at the genus level: *5-7 N15*, *CF231*, *Oscillospira*, *Paludibacter,* and *Akkermansia* (*p* > 0.05 in all cases), except that *rc4–4* was lower in the CON and SMFP groups than in the EML group (*p* = 0.02). There were no significant differences in the richness estimate and diversity indices between the groups (*p* > 0.16), and the different diets did not significantly influence most selected fecal bacterial species (*p* > 0.06), except for *Ruminococcus albus*, which was higher in the EML group (*p* < 0.01) and *Streptococcus bovis*, which was lower in the CON group (*p* < 0.01) relative to the other groups.

**Conclusions:**

In conclusion, diets supplemented with EML and SMFP have little influence on the fecal bacterial community composition in finishing steers.

**Electronic supplementary material:**

The online version of this article (doi:10.1186/s12866-017-1011-9) contains supplementary material, which is available to authorized users.

## Background

With the increased demand for animal production in many developing countries, including China, there is an obvious demand for sufficient and inexpensive livestock feed. Several reports have shown that agricultural byproducts and fruit residue can be used to replace cereal-based concentrates as livestock feed without negatively affecting animal production performance. Previous studies have compared the dry matter digestibility of several grape pomace varieties in cows, sheep, and goats in vitro [1–3], and in vivo experiments have investigated feeds such as apple pomace silage [4], which is used for sheep [5], and potato pomace, which is used as protein supplement for growing lambs [6]. Mulberry pomace, which is a byproduct of mulberry juice, consists mainly of peels and stems and accounts for approximately 8% of the fresh weight of the mulberry [7–10]. In our previous study, we found that ensiled mulberry leaves (EML) contains 19.8% crude protein (CP), 15.6% water soluble carbohydrates (WSC), and 51.5% neutral detergent fiber (NDF), and sun-dried mulberry fruit pomace (SMFP) contains 21.8% CP, 20.8% WSC, and 49.1% NDF [11]. Furthermore, both EML and SMFP can be used in finishing steers diets without impairing their productive performance and carcass characteristics [12, 13].

However, little is known about how EML and SMFP could influence the fecal microbial community structure. The intestinal microbiome of cattle plays a critical role not only in animal health and productivity but also in food safety and environmental protection [14]. A previous survey suggested that bovine fecal bacterial community structures can dramatically differ at the phylum and family levels depending on the animal feed. Furthermore, the feeding operation has been reported to be a more important determinant of the cattle microbiome than the geographic location of the feedlot [15, 16]. Our hypothesis is that EML and SMFP can be used in the diets for finishing steers without affecting the fecal bacterial community composition, and harmless to animal health and food safety. To validate this assumption, we used sequencing analysis to examine the effects of EML and SMFP on the fecal bacterial communities in cattle fed these byproducts.

## Methods

### Animals and diets

The animals in this study were handled in strict accordance with the Regulations for Laboratory Animals of Beijing. The protocol was approved by the Animal Welfare Committee of China Agricultural University (Permit No.DK1008). Experiments were performed in accordance with the Regulations for the Administration of Affairs Concerning Experimental Animals (The State Science and Technology Commission of P. R. China, 1988).

This study was part of a larger experimental trial investigating the effects of EML and SMFP on growth performance, ruminal fermentation, blood biochemical parameters, and carcass characteristics of finishing steers [11–13]. The experiment was done in the experimental base of China Agricultural University. A total of 51 healthy Simmental crossbred steers weighing 357.06 ± 16.5 kg (average age, 15 months) were divided into three treatment groups (*n* = 17) and each group was fed a different diet. The control group (CON) received standard TMR, the EML received a standard diet containing EML, and the sun-dried mulberry fruit pomace group (SMFP) received a standard diet containing SMFP (Additional file 1: Table S1). The ingredients and nutrient composition of each diet are provided in Additional file 1: Table S1 and in our previous report [11]. The animals were fed twice daily at approximately 0800 and 1700 h to meet the NRC recommendations and had ad libitum access to water. To investigate the effects of EML and SMFP on the fecal bacterial community, 4 steers were selected randomly in every treatment, and totally 12 steers were selected.

### Fecal sampling

At the end of the experiment, 30–50 g fecal samples were obtained aseptically from cattle rectums with a new palpation sleeve used for each sample. The fecal samples were quickly sealed in 50-mL conical tubes. Sampling was accomplished as quickly as possible. The samples were then frozen in liquid nitrogen prior to storage at −80 °C until genomic DNA was extracted.

### DNA extraction and sequencing

Total genomic DNA was extracted from 0.2 g of frozen fecal sample using a fecal DNA extraction toolkit (Tiangen Biotech Co., Beijing, China) combining a bead-beat with an oscillator (Precellys 24, Bertin Technology, Montigny-le-Bretonneux, France) plus column method [17]. The rotating speed of the oscillator was 5500 rpm with two circulations and 30 s per circulation. RNA was digested with 50 μg RNase A, and DNA was subsequently cleaned up and eluted in 50 μL of EB. The DNA samples were quantified using a NanoDrop 2000 spectrophotometer (Thermo Fisher Scientific Inc., Wilmington, DE). Subsequently, DNA samples were diluted in TE buffer to obtain a concentration of 5 mM. Sequencing was conducted on an Illumina MiSeq platform v2 2 × 250-bp paired-end protocol yielding paired-end reads. Briefly, DNA was amplified using the universal eubacterial primer set (515f: 5′-GTG CCA GCM GCC GCG GTA A-3′, 806r: 5′-XXX XXX GGA CTA CHV CCC TWT CTA AT-3′), which targets the hypervariable V4 region of the *16S* rRNA gene, with the reverse primer containing a 6-bp error-correcting barcode unique to each sample [18]. Amplification was performed using Phusion High-Fidelity PCR Mastermix (New England Biolabs [Beijing] Ltd., China). The PCR conditions consisted of 3 min at 94 °C, followed by 35 cycles of 15 s at 94 °C, 15 s at 58 °C, 10 s at 68 °C, and a final elongation step of 30 s at 68 °C. Amplicons were selected on 2% agarose gels on E-Gel® Size Select™ Agarose Gel and then purified with Agencourt® AMPure® XP Reagent. The purified DNA was quantified with Quant-iTTM Technology (Life Technologies, Inc.) applying Quant-iTTM dsDNA Broad-Range Assay Kit. An Agilent 2100 Bioanalyzer™ with a High-Sensitivity DNA Kit (Agilent Technologies, Inc., Santa Clara, CA) was used to analyze library sizes and molar concentrations. Emulsion PCR was performed using the Ion OneTouch™ 200 Template Kit v2 DL (Life Technologies, Inc.) according to the manufacturer’s instructions.

### Sequence analysis

The Illumina MiSeq sequencing data were analyzed using QIIME software (version 1.7.0). Filters were applied to sequences prior to phylogenetic analysis. Depending upon the appropriate fragment size for V4 PCR (150–200 bp), bases after position 200 were trimmed and reads shorter than 150 bp were removed. Reads with a quality score of <25 were removed with the NGSQC Toolkit, and only sequences without any ambiguous characters were included in the analysis. To calculate the downstream diversity (alpha and beta diversity), all the samples were subsampled to an equal size of 100,000 reads before comparison of the bacterial communities. The sequences were clustered into operational taxonomic units (OTUs) at the 97% sequence identity level, and the most abundant sequence of each OTU was chosen as a representative. Based on the OTUs, rarefaction curve and alpha diversity indices (i.e., abundance-based coverage estimator [ACE], and the Chao1, Shannon, and Simpson estimators) were developed. The jackknifed beta diversity included the calculated unweighted and weighted Unifrac distances, which were visualized by principal coordinate analysis (PCoA) [19]. A two-dimensional hierarchical clustering heatmap was drawn based on the number of reads of each OTU using R software (version 3.2.4).

### Quantitative real-time PCR

Quantitative real-time PCR with specific primers and probes was used to determine the population size of thirteen major fecal bacteria. All the qPCR assays were quantified using SYBR Green PCR RealMaster Mix (Tiangen Biotech, co., LTD, China) on an ABI-7300 Prism real-time PCR instrument (ABI, Foster City, CA). The following PCR program was used: one cycle of 95 °C for 15 min (initial denaturation), 40 cycles of 95 °C for 15 s (denaturation) and 60 °C for 32 s (annealing). Following qPCR, the products of amplification were confirmed by agarose gel (1.2%) electrophoresis. To minimize variations, all real-time PCR assays were performed in triplicate. The abundance of fecal microbes was recorded and multiplied by the dilution factor to determine the total number of target microbes per gram (wet weight).

### Statistical analysis

The read number, sample coverage, unique OTUs, sample richness (Chao1 and ACE), and sample diversity (Shannon-Wiener and Simpson’s indices) were compared with the general linear model (GLM) and one-way analysis of variance (ANOVA) using SAS 9.0 (SAS Institute, Cary, NC). Abundance of phylum and genus was determined to assess the effects of EML and SMFP supplementation. Absolute abundance of microbes was expressed as copies of 16S rRNA genes per gram (wet weight). Statistical significance was set at *p* ≤ 0.05.

## Results

### Sequencing and general bacterial community composition

**Fig. 1:**
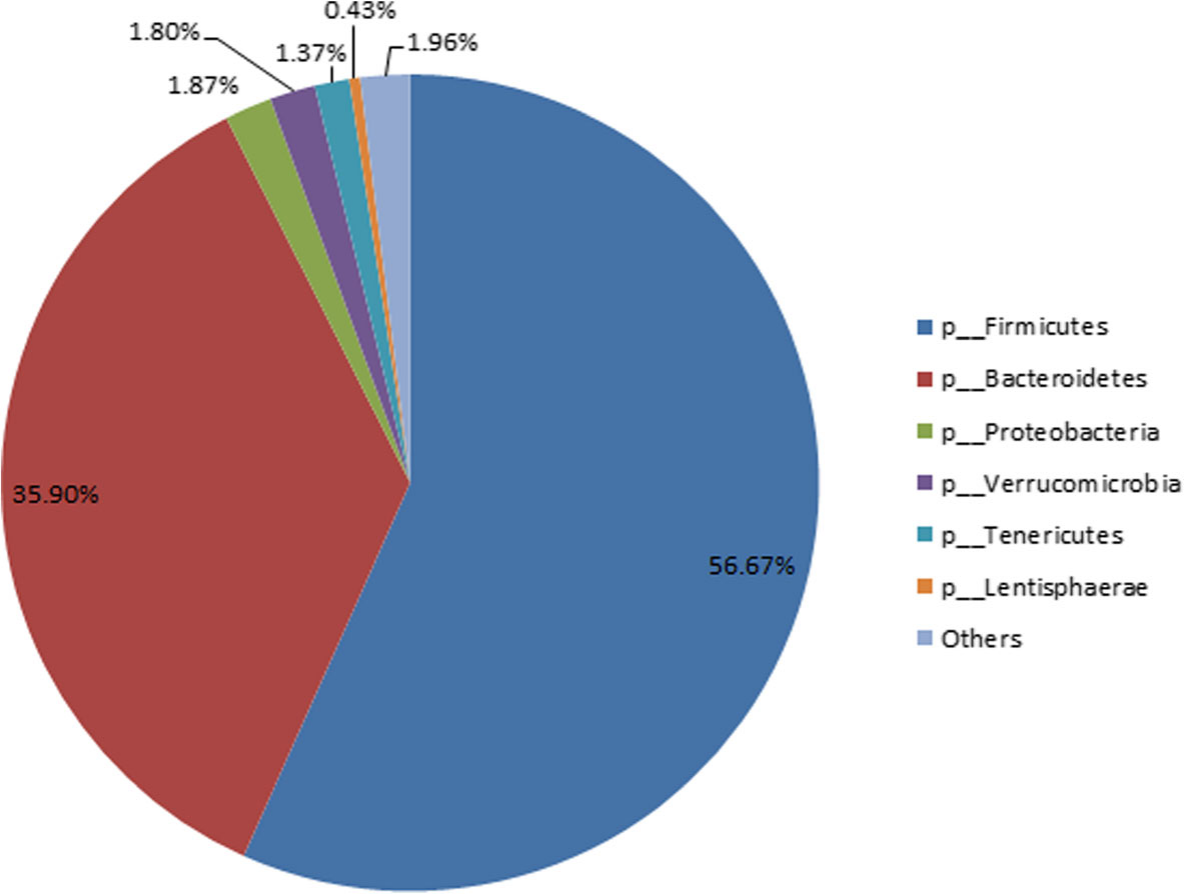
Average relative abundance of fecal bacteria in steers fed different diets

**Fig. 2:**
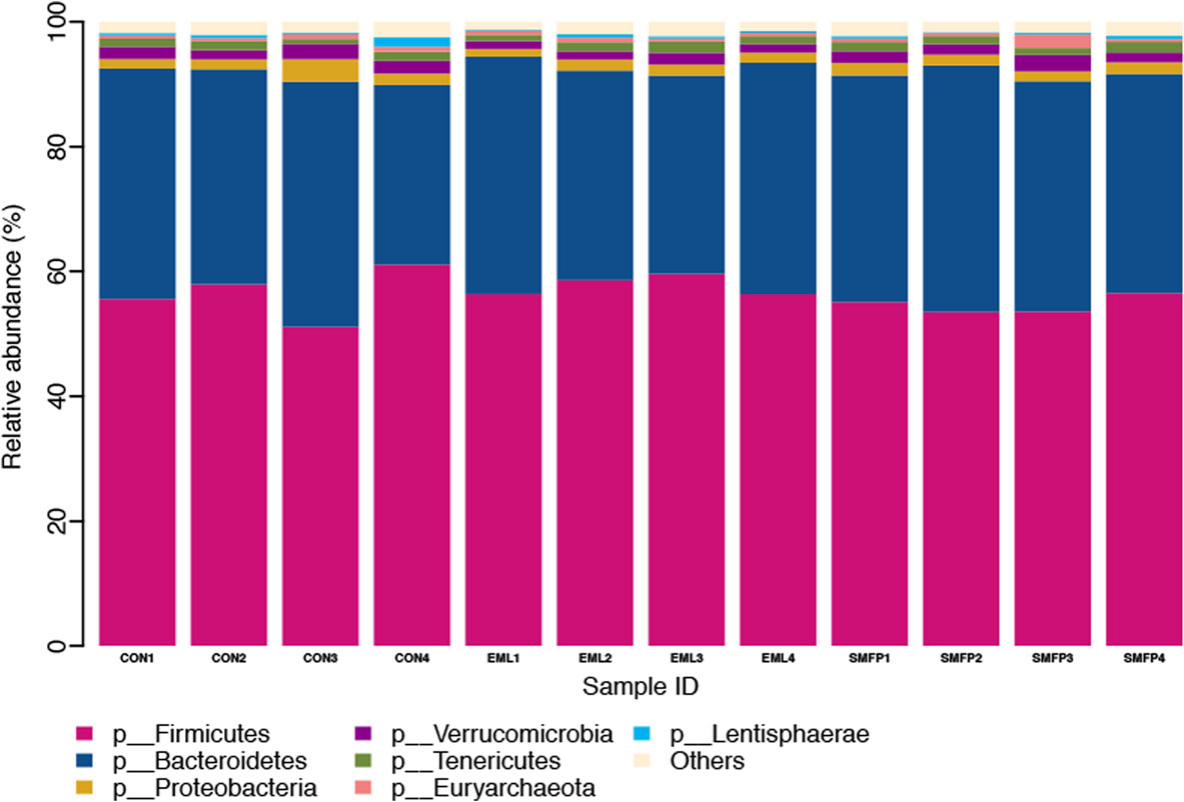
Bar chart showing the relative abundance of fecal bacterial composition at the phylum level

A total of 2,245,345 quality-checked sequences were obtained from the 12 samples, and 133,780–249,943 sequences were returned for each sample. After OTU picking and chimera checking, a total of 17,761 OTUs were calculated for the 12 samples at 3% dissimilarity. The average OTUs for the CON, EML, and SMFP group were 7498, 6933, and 6503 per sample, respectively. After normalization to 100,000 reads, richness estimates and diversity indices were developed (Table 1). Good’s coverage for each sample ranged from 0.9679 to 0.9853, with a mean value of 0.9748 for all samples. The rarefaction curve (Additional file 1: Figure S1) indicated that a reasonable number of individual samples had been obtained. The most abundant phyla for all 12 samples were *Firmicutes* (56.67%), *Bacteroidetes* (35.90%), *Proteobacteria* (1.87%), *Verrucomicrobia* (1.80%), and *Tenericutes* (1.37%; Table 2). The minor phyla, accounting for less than 1% of the bacterial communities was *Lentisphaerae* (0.43%), and the other known phyla account for 1.96% of the total sequences. Figure 1 shows the average relative abundance of bacterial phyla in the fecal samples. Figure 2 illustrates the community composition of individual samples in the different treatments. The bar chart illustrates seven bacterial phyla identified in the fecal samples with high relative abundance. A total of 282 genera were detected from the 12 fecal samples. Known genera with high abundance (more than 0.1%) belonged primarily to the phyla *Bacteroidetes* and *Firmicutes*, but they all had low abundance, and only five of these genera accounted for >1% of total sequences. These five “predominant” genera were *5-7 N15* (5.91%), *CF231* (2.49%), *Oscillospira* (2.33%), *Paludibacter* (1.23%), and *Akkermansia* (1.11%). Minor genera such as *Dorea*, *rc4–4*, *Prevotella*, *Methanobrevibacter,* and *Campylobacter* accounted for 0.98, 0.93, 0.75, 0.57, and 0.19% of the total sequences, respectively. The other known genera accounting for 4.23 and 79.23% of the sequences represent strains that were unclassified at the genus level (Table 3).

**Table 1:** Dietary treatments for unique OTUs, richness estimates, and diversity indices within the fecal content

Item	Treatment^a^			SEM^b^	*p* value
	CON	EML	SMFP		
SeqsNum	166,882.30	184,494.30	178,779.30	19,052.18	0.8
OTUsNum	7498.00	6932.75	6503.25	610.56	0.54
EvenSeqsNum	100,000	100,000	100,000	-	-
EvenOTUsNum	5866.00	5253.00	5012.75	346.74	0.25
PD-whole-tree	214.29	196.79	191.16	10.17	0.29
Observed species	5866.00	5253.00	5012.75	346.74	0.25
Good’s coverage	0.97	0.98	0.98	0.00	0.22
Richness estimate					
Chao1	11,386.85	9787.32	8929.23	834.02	0.16
ACE	11,616.52	9930.3	8982.59	937.71	0.19
Diversity indices					
Simpson	1.00	1.00	1.00	0.00	0.45
Shannon	9.70	9.58	9.50	0.10	0.37

^a^ *CON* Control (*n* = 4), *EML* Ensiled mulberry leaves (*n* = 4), *SMFP* Sun-dried mulberry fruit pomace (*n* = 4)

^b^SEM: standard error of the mean

**Table 2:** Effect of EML and SMFP diet on changes in phyla (as a percentage of the total number of sequences) in the fecal bacterial community

Phylum	Experimental diet^a^			SEM^b^	*p* value
	CON	EML	SMFP		
*Firmicutes*	56.79	58.06	55.18	1.39	0.28
*Bacteroidetes*	35.09	35.34	37.28	1.6	0.63
*Proteobacteria*	2.16	1.62	1.83	0.28	0.46
*Verrucomicrobia*	1.99	1.47	1.94	0.18	0.17
*Tenericutes*	1.27	1.41	1.42	0.15	0.75
*Lentisphaerae*	0.63	0.32	0.32	0.18	0.41
Others	2.06	1.78	2.03	0.18	0.59
*Firmicutes:Bacteroidetes*	1.61	1.64	1.48	0.12	0.54

^a^ *CON* Control (*n* = 4), *EML* Ensiled mulberry leaves (*n* = 4), *SMFP* Sun-dried mulberry fruit pomace (*n* = 4)

^b^ *SEM* Standard error of the mean

Different letters in the same row indicate a significant difference between values within the row (*p* < 0.05)

**Table 3:** Effect of EML and SMFP on changes in the genus composition (as a percentage of the total number of sequences) in the fecal bacterial community

Genus	Experimental diet^1^			SEM^2^	*p* value
	CON	EML	SMFP		
*5-7 N15*	5.27	5.95	6.51	0.45	0.21
*CF231*	2.36	2.16	2.95	0.59	0.62
*Oscillospira*	2.31	2.35	2.33	0.07	0.90
*Paludibacter*	1.12	1.18	1.37	0.12	0.33
*Akkermansia*	1.14	0.91	1.28	0.18	0.37
*Dorea*	0.96	1.03	0.95	0.08	0.78
*rc4–4*	0.88^b^	1.21^a^	0.71^b^	0.10	0.01
*[Prevotella]*	0.57	0.65	1.02	0.15	0.12
*Methanobrevibacter*	0.46	0.47	0.77	0.26	0.65
*Campylobacter*	0.50	0.01	0.07	0.27	0.41
Others	4.02	4.29	4.55	0.23	0.30
Unknown	80.40	79.79	77.48	1.53	0.40

^1^ *CON* Control (*n* = 4), *EML* Ensiled mulberry leaves (*n* = 4), *SMFP* Sun-dried mulberry fruit pomace (*n* = 4)

^2^ *SEM* Standard error of the mean

Different letters in the same row indicate a significant difference between values within the row (*p* < 0.05)

### Changes in the fecal microbiota by the EML and SMFP diets

**Fig. 3:**
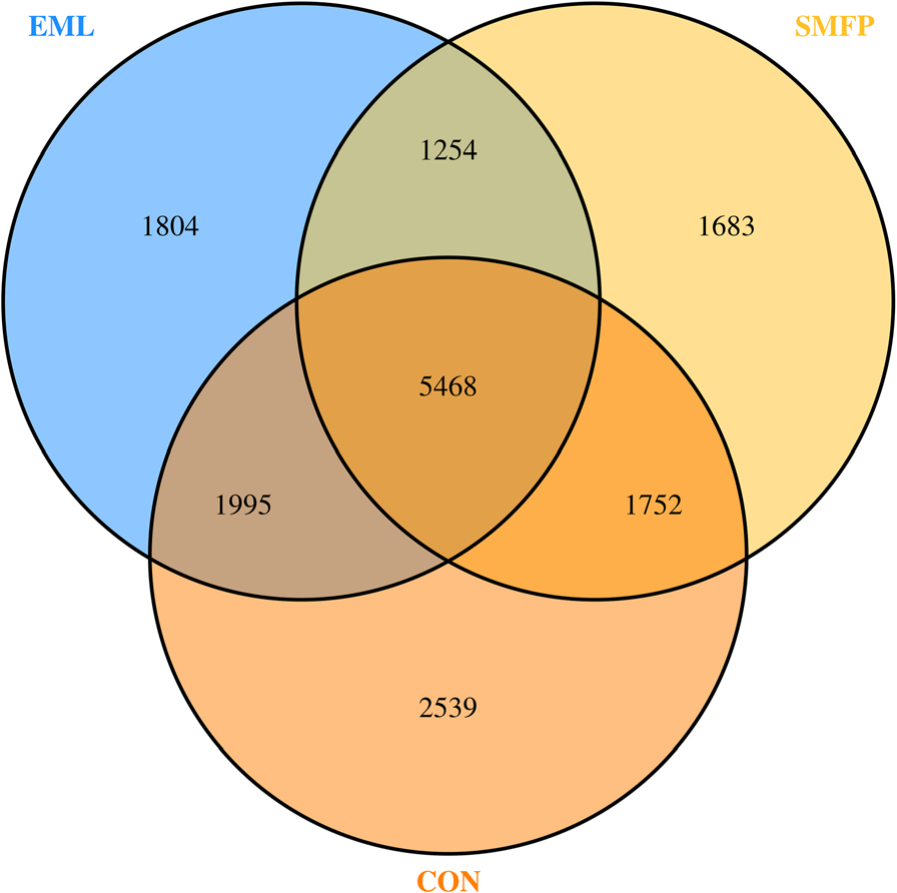
Venn diagram illustrating the distribution of unique, shared, and common phylotypes among *16S* rRNA gene libraries in the three groups

Table 1 shows the OTUs, richness estimate, and diversity indices of all the 12 fecal samples across the three groups. The results of the Illumina MiSeq sequencing analyses revealed that addition of EML and SMFP to the feed did not affect the alpha or beta diversity of fecal bacteria (Table 1). The ACE and Chao1 indices were calculated to compare species richness by estimating the minimum number of unique OTUs for each sample. The Chao1 (*p* = 0.16) and ACE (*p* = 0.19) values indicated that species richness was the highest in the CON group; however, there was no significant difference in the species richness between groups. Similarly, the Shannon-Wiener (*p* = 0.37) and Simpson (*p* = 0.45) indices both showed that the bacterial community exhibited considerable diversity, but with no differences between groups. The Venn diagram in Fig. 3 illustrates the distribution of all unique, shared, and common phylotypes among *16S* rRNA gene libraries prepared from the fecal samples from the three groups. An examination of the phylotypes showed that there were 5468 phylotypes in the fecal communities in all three groups (Fig. 3). Furthermore, steers from the CON group were found to have more phylotypes over the entire course of the experiment (11,754 OTU) compared to those from the EML and SMFP groups (10,521 and 10,157 OUT, respectively), which was in line with the richness estimate results. A thermal double dendrogram of the 50 most abundant bacterial OTUs (Additional file 1: Figure S2) demonstrated that samples in the same treatment could not be easily grouped, indicating that there were considerable similarities in the fecal bacterial communities of the different treatment groups.

The distribution was based on a 3% species cutoff. The *16S* gene libraries were prepared from fecal samples obtained from cattle fed the CON (*n* = 4), EML (*n* = 4), and SMFP (*n* = 4) diets.

To assess the potential effect of EML and SMFP diets on fecal bacterial communities, sequencing datasets from individual groups of steers were examined (Table 2). Both diets had no influence on the proportion of *Firmicutes*, which is a major gram-positive phylum in the fecal samples (*p* = 0.28). Similarly, the proportion of the major gram-negative phylum *Bacteroidetes* was also unchanged by the EML and SMFP diets (*p* = 0.63). There were no statistically significant differences in the less abundant bacterial phyla among the three groups (*p* > 0.10; Table 2). To evaluate the effects of the EML and SMFP diets on the fecal bacterial community composition, genera with an abundance of >0.3% were selected. Most of the selected genera did not show a remarkable difference among the three groups (*p* > 0.05), except for *rc4–4* (*p* = 0.01), which was increased in the EML group (Table 3). The above data indicated that the bacterial community did not significantly differ between the three groups.

**Fig. 4:**
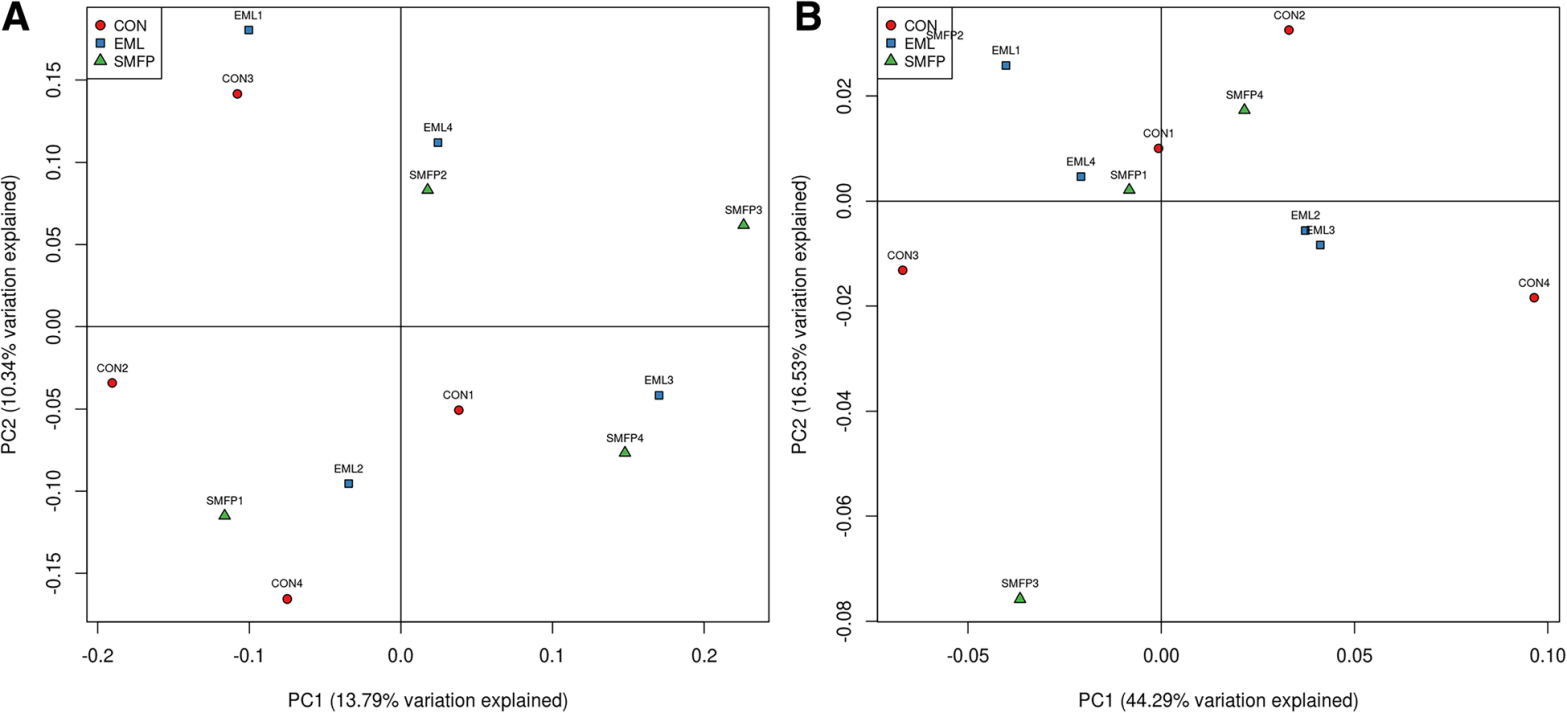
Weighted (**a**) and unweighted (**b**) principal coordinate analysis (PCoA) illustrating relationships among fecal bacterial populations in steers fed different diets

Figure 4 presents a comparison among the 12 samples by PCoA. The weighted and unweighted PCoA indicated that the relative abundances of bacterial populations were not affected by diets All the sample points were equally distributed in the coordinates (weighted and unweighted); meanwhile, the three clusters could not be obviously separated from each other, i.e., the bacterial communities of the three groups did not significantly differ. The difference between the groups was less obvious, and the CON group was not distinguishable from the EML and SMFP groups.

Weighted PCoA was based on the relative abundance of bacterial communities, while unweighted PCoA was based on the uniqueness of the bacterial communities. CON, control group (*n* = 4); EML, ensiled mulberry leaves (*n* = 4); SMFP, sun-dried mulberry fruit pomace (*n* = 4).

### Proportion of selected bacteria in feces

Table 4 presents the results of the real-time qPCR analyses of 13 selected fecal bacteria. As shown in the table, EML and SMFP affected the composition of fecal bacteria, as reflected by the specific species. Compared with the CON group, the populations of *Ruminococcus albus* (*p* = 0.0015) and *Streptococcus bovis* (*p* = 0.0086) were found to be significantly increased in the EML and SMFP groups, while the *Selenomonas ruminantium* (*p* = 0.0591), *Succinivibrio dextrinosolvens* (*p* = 0.0652), and *Butyrivibrio fibrisolvens* (*p* = 0.0702) populations were increased in these two groups, although the difference in this case was not statistically significant. The remaining eight examined bacterial targets showed no difference between the different treatment groups.

**Table 4:** Fecal bacterial abundance in finishing steers fed a total mixed ration supplemented with EML and SMFP

Bacteria	Experimental diet^1^			SEM^2^	*P* value
	CON	EML	SMFP		
*Fibrobacter succinogenes*	0.0268	0.0280	0.0361	0.0149	0.8933
*Prevotella bryantii*	0.0006	0.0068	0.0007	0.0032	0.3577
*Prevotella ruminicola*	0.0005	0.0089	0.0008	0.0049	0.4510
*Ruminococcus flavefaciens*	0.1069	0.1211	0.1735	0.0615	0.7336
*Selenomonas ruminantium*	0.0725	0.1424	0.1277	0.0170	0.0591
*Succinivibrio dextrinosolvens*	0.0993	3.2201	3.9093	0.9618	0.0652
*Butyrivibrio fibrisolvens*	0.0001	0.0170	0.0179	0.0049	0.0702
*Eubacterium ruminatium*	0.0000	0.0047	0.0020	0.0016	0.2129
*Megasphaera elsdenii*	0.0001	0.0105	0.0060	0.0031	0.1337
*Prevotella brevis*	0.0000	0.0021	0.0009	0.0007	0.1935
*Ruminobacter amylophilus*	0.0000	0.0484	0.0038	0.0269	0.4220
*Ruminococcus albus*	0.0114^b^	1.7266^a^	0.5975^b^	0.1804	0.0015
*Streptococcus bovis*	0.0034^b^	0.2918^a^	0.2086^a^	0.0435	0.0086

^1^ *CON* Control (*n* = 4), *EML* Ensiled mulberry leaves (*n* = 4), *SMFP* sun-dried mulberry fruit pomace (*n* = 4)

^2^ *SEM* Standard error of the mean

Population sizes are expressed as percentages of the *16S* rRNA gene copy number of the total bacteria

Different letters in the same row indicate a significant difference between values within the row (*p* < 0.05)

## Discussion

The gut bacterial composition of animals is influenced by many factors, and their primary function is to metabolize undigested carbohydrates absorbed by the upper gut, resulting in the production of organic acids, gases, and short-chain fatty acids [20–22]. The bacterial composition of the intestine has a significant impact on the growth and health of cattle and is associated with fecal contamination of environmental sources and human illness via foodborne pathogens [23–26]. Many DNA sequencing studies that have analyzed the feces of beef and dairy cattle from a variety of geographical locations and different management practices have identified a core set of three phyla across all cattle. These three phyla, in order of relative abundance are *Firmicutes*, *Bacteroidetes*, and *Proteobacteria* [27]. This report is in agreement with the dominant phyla reported in many other studies of mammalian gut microbiota [28–30] as well as in our present study. *Firmicutes* and *Bacteroidetes* were dominant phyla in all fecal samples. The ratio of *Firmicutes* to *Bacteroidetes* has been shown to affect energy harvesting and body fat in humans and mice, and a decreased amount of *Bacteroidetes* in the microbiota was correlated with increased fat in blood and tissue [31, 32]. Research has demonstrated that there is a relationship between dietary efficiency and the diversity of gastrointestinal bacterial populations. Studies on mice have shown that genetically obese mice had a greater population of bacteria from *Firmicutes* than *Bacteroidetes* when compared with genetically lean mice [31, 33]. In the present study, we found no statistically significant differences in the *Firmicutes*:*Bacteroidetes* ratio in the fecal population, but we did observe a trend for smaller *Firmicutes*:*Bacteroidetes* ratios in the SMFP group. Compared to our previous study [11] involving the analysis of fat content in beef, the fat content of beef was found to be significantly lower in the SMFP group, indicating that certain fat-decreasing components may exist within SMFP products; this result may be due to the altered *Firmicutes*:*Bacteroidetes* ratio in this group.

At the genus level, more than 1% of the total sequences belonged to *5-7 N15*, *CF231*, *Oscillospira*, *Paludibacter,* and *Akkermansia*. This is different from the results of other studies where *Prevotella*, *Ruminococcus*, *Butyrivibrio*, *Succiniclasticum*, etc., were reported as the dominant genera in the fecal microbial communities in cattle [34–36]. This distinction indicates that the fecal microbial community structure in cattle is greatly affected by diet [37]. Durso et al. indicated that *Pervotella* was commonly found in cattle feces and associated with dietary differences [38]. *Prevotella* is believed to play an important role in starch degradation. There are many non-structural carbohydrates and starches present in mulberry fruit pomace [11], which can potentially promote the activity or proliferation of *Prevotella*. While there was only a slight difference in the population of *Prevotella* among the three treatment groups, the *Prevotella* population was slightly increased in the SMFP group compared to the other two groups. *Oscillospira* seems to be positively correlated with starch content. In humans, *Oscillospira* was increased in diets containing resistant starch [39]. Cattle fed diets with a high starch content have been shown to have increased bypass of starch from the rumen [40] and *Oscillospira* in cattle feces may be associated with the high levels of starch bypass. Ruminal *Oscillospira* are positively correlated with diets rich in forage [41]. While there were no significant differences in the bacterial abundance between the three groups in our result, the addition of supplement at low level may be responsible for the lack of change in fecal bacterial abundance of *Oscillospira*.

Many previous studies investigating the microbial community in the digestive system of ruminants have focused on the rumen, which is the largest and most important compartment of the stomach in ruminants [42–45]. However, as the large intestine, especially the caecum and colon, is an important organ for nutrition supply and absorption in ruminants, it has received considerable research attention [46]. Some studies have suggested that the large intestine provides an active fermentation condition similar to the reticulum-rumen. Through quantitative PCR, we were able to illustrate changes in individual target species, and these species are among the most commonly researched fecal microbial species. We recognized *Fibrobacter succinogenes*, *Ruminococcus flavefaciens*, and *R. albus* as the major cellulolytic bacterial species, while *S. ruminantium* is the dominant hemicellulose-degrading bacteria [47], which is highly oriented towards fiber degradation. All of the above species are important in the intestine environment, as they are responsible for the digestion of cellulose and hemicellulose in the intestine of ruminants. Cellulose and hemicellulose are mainly digested in the large intestine of cattle, accounting for 18–27% and 20–40% of the total digested cellulose and hemicellulose, respectively [48]. It is reasonable that the large intestine has a strong ability to digest cellulose and hemicellulose, as fecal bacteria in the large intestine produce special cellulolytic enzymes that could hydrolyze pentosans and hemicellulose. In this report, the population of *R. albus* was significantly increased in the EML group (*p* = 0.0015), but the other cellulolytic bacteria were not influenced by the EML and SMFP diets. This finding indicates that supplementing the diet with EML may positively influence cellulose degradation. Amylolytic species of fecal bacteria, including *Prevotella bryantii*, *Prevotella ruminicola*, *S. bovis*, and *Ruminobacter amylophilus* in the present study, use intracellular amylase to hydrolyze starch to glucose, maltose, maltotriose, and maltotetraose [49]. Our result showed that the abundance of *P. bryantii* and *P. ruminicola* had an increasing tendency in the EML group, and both the EML and SMFP diets increased the abundance of *R. amylophilus*. The abundance of *S. bovis* was significantly influenced by both the EML and SMFP supplements. Previous studies indicated that the large intestine of cattle contain a small quantity of starch, and that the large intestine is an important organ for digesting glucose and soluble glucosides [50], indicating that EML and SMFP supplementation can have a positive influence on starch degradation in the upper gut and consequently increase the glucose content of the intestine. In our previous research [11], in vitro gas production at 48 h was significantly higher in the SMFP treatment than in the CON treatment group, indicating that the SMFP diet may produce more fermentable glucose. Most fecal microorganisms have some proteolytic activity, of which *Butyrivibrio fibrisolvens*can be regarded as one of highest proteolytic activity strain. Based on the increasing tendency of *Butyrivibrio dextrinosolvens* in the EML and SMFP groups relative to the control group, we proposed that EML and SMFP supplementation could promote the utilization of protein resources by gut microorganisms.

## Conclusion

In this study, we compared the community composition of fecal microbiota in cattle fed a standard TMR and in those fed with modified TMR diets in which corn grain and cotton seed meal were partially replaced by EML or SMFP. The results of this study demonstrate that addition of EML and SMFP to the TMR only slightly influence the fecal microbial community structure in finishing steers.

## Additional file

Additional file 1: Figure S1. Rarefaction curve for each sample with a cutoff level of 0.03 (*n* = 12). **Figure S2.** Double dendrogram of bacteria present in the rumen of steers fed different diets. **Table S1.** Ingredients and nutrient composition of the experimental diets. (DOCX 323 kb)

## Abbreviations

ACE: Abundance-based coverage estimator
CON: Control
CP: Crude protein
EML: Ensiled mulberry leaves
NDF: Neutral detergent fiber
OUTs: Operational taxonomic units
PCoA: Principal coordinate analysis
PCR: Polymerase chain reaction
rt-PCR: Real time - polymerase chain reaction
SEM: Standard error of mean
SMFP: Sun-dried mulberry fruit pomace
TMR: Total mixed ration
WSC: Water soluble carbohydrates
