# Supplementary material for: Effect of ensiled mulberry leaves and sun-dried mulberry fruit pomace on the fecal bacterial community composition in finishing steers
Source: BMC Microbiol. 2017 Apr 21;17:97. doi: 10.1186/s12866-017-1011-9 (PMC5401608; doi:10.1186/s12866-017-1011-9)
Supplement: Additional file 1: Figure S1. — Rarefaction curve for each sample with a cutoff level of 0.03 (n = 12). Figure S2. Double dendrogram of bacteria present in the rumen of steers fed different diets. Table S1. Ingredients and nutrient composition of the experimental diets. (DOCX 323 kb) [file 12866_2017_1011_MOESM1_ESM.docx]

Supplementary Material

**Effect of Ensiled Mulberry Leaves and Sun-Dried Mulberry Fruit Pomace on the Fecal Bacterial Community Composition in Finishing Steers**

**Yan Li^1^, Qingxiang Meng^1^****, Bo Zhou^1^ and Zhenming Zhou^1,*^**

**^*^Correspondence:** Corresponding Author: [zhouzm@cau.edu.cn](mailto:zhouzm@cau.edu.cn)

**1 Supplementary Data**

Table 1

Table 1

Table 2

Table 3

Figure 1 and figure 3

**2 Supplementary Figures and Tables**

**2.1 Supplementary Figures**


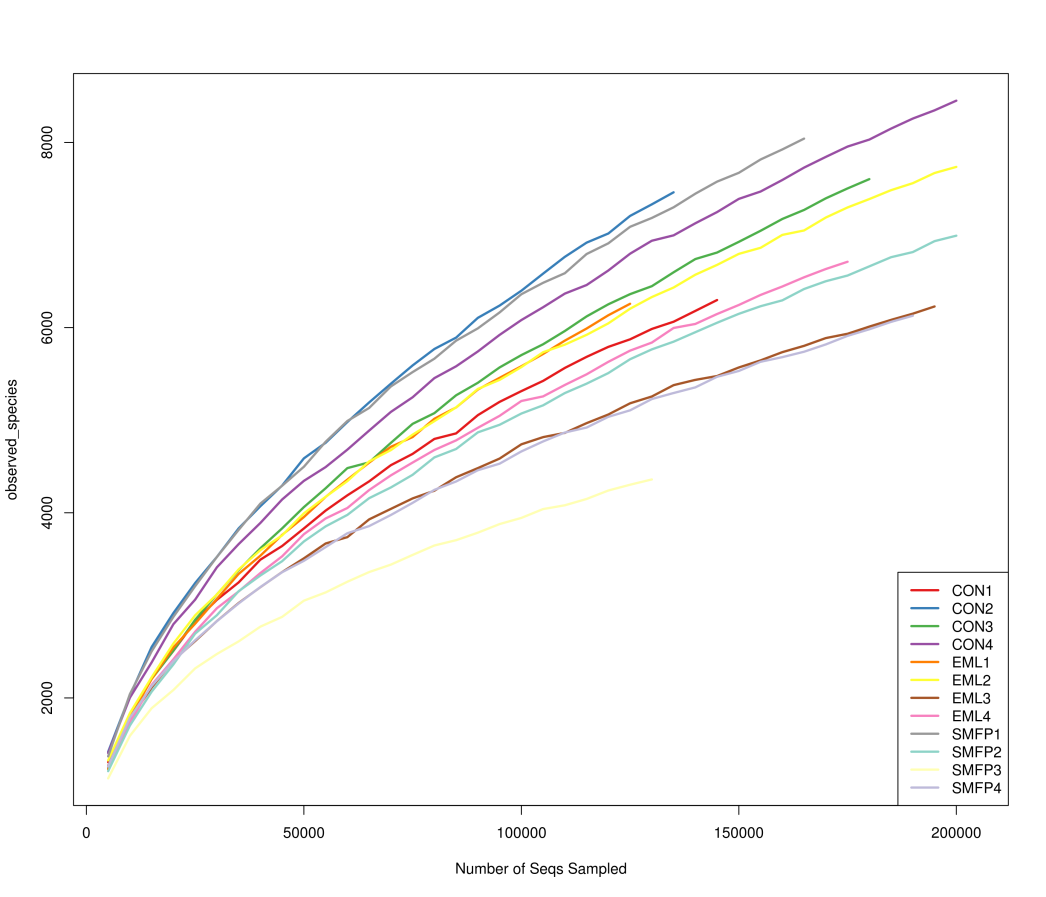


**Supplementary Figure 1.** Rarefaction curve for each sample with a cutoff level of 0.03 (n = 12).

**
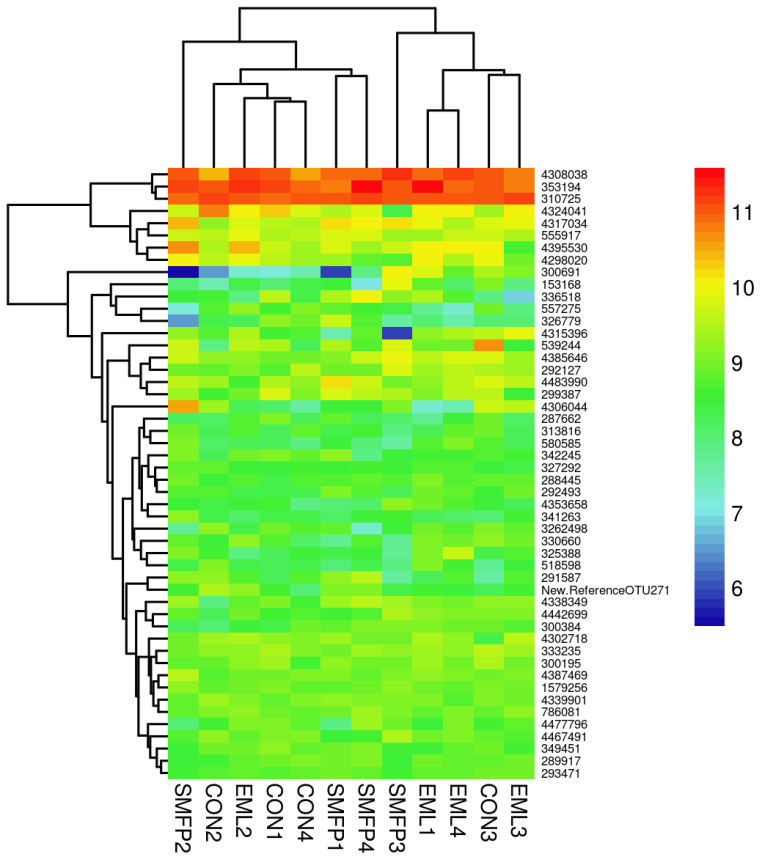
**

**Supplementary Figure 2.** Double dendrogram of bacteria present in the rumen of steers fed different diets.

**2.2 Supplementary Tables**

**Supplementary Table 1.** Ingredients and nutrient composition of the experimental diets.

| **Item** | **Experimental diet^1^** | | |
| --- | --- | --- | --- |
|  | CON | EML | SMFP |
| **Ingredient composition, % (DM basis)** | | | |
| **Corn silage** | 40.0 | 40.0 | 40.0 |
| **Brewer’s grain** | 20.0 | 20.0 | 20.0 |
| **Corn** | 30.2 | 27.0 | 24.5 |
| **Cotton seed meal** | 7.8 | 3.0 | 7.2 |
| **Ensiled mulberry leaves** | 0.0 | 8.0 | 0.0 |
| **Sun-dried mulberry fruit pomace** | 0.0 | 0.0 | 6.3 |
| **Limestone powder** | 0.7 | 0.7 | 0.7 |
| **Calcium hydrogen phosphate** | 0.1 | 0.1 | 0.1 |
| **Sodium bicarbonate** | 0.5 | 0.5 | 0.5 |
| **Salt** | 0.5 | 0.5 | 0.5 |
| **Premix^2^** | 0.2 | 0.2 | 0.2 |
| **Nutrient composition, DM basis** | | | |
| **ME (Mcal/kg)^3^** | 2.69 | 2.69 | 2.70 |
| **CP (%)** | 14.01 | 14.06 | 13.98 |
| **Ca (%)** | 0.56 | 0.57 | 0.55 |
| **P (%)** | 0.33 | 0.33 | 0.33 |
| **ADF (%)** | 21.42 | 22.56 | 21.51 |

^1^CON = control (n = 4); EML = ensiled mulberry leaves (n = 4); SMFP = sun-dried mulberry fruit pomace (n = 4).

^2^Supplied per kilogram of dietary DM: 15 mg of Cu, 65 mg of Zn, 28 mg of Mn, 0.7 mg of I, 0.2 mg of Co, 0.3 mg of Se, 6000 IU of vitamin A, 600 IU of vitamin D, and 47 IU of vitamin E.

^3^ME = metabolizable energy; CP = crude protein; ADF = acid detergent fiber.
